# Epidemiological Study of the Occurrence of Typhus Group *Rickettsia* Natural Infection in Domiciliated Dogs from a Rural Community in South-Eastern Mexico

**DOI:** 10.3390/ani12202885

**Published:** 2022-10-21

**Authors:** Marco Torres-Castro, Enrique Reyes-Novelo, Manuel Bolio-González, Cesar Lugo-Caballero, Karla Dzul-Rosado, Pablo Colunga-Salas, Sokani Sánchez-Montes, Henry Noh-Pech, Fernando I. Puerto, Roger Iván Rodríguez-Vivas

**Affiliations:** 1 Laboratorio de Enfermedades Emergentes y Reemergentes, Centro de Investigaciones Regionales “Dr. Hideyo Noguchi”, Universidad Autónoma de Yucatán, Mérida 97000, Mexico; 2 Laboratorio de Zoonosis y otras Enfermedades Transmitidas por Vector, Centro de Investigaciones Regionales “Dr. Hideyo Noguchi”, Universidad Autónoma de Yucatán, Mérida 97000, Mexico; 3 Facultad de Medicina Veterinaria y Zootecnia, Campus de Ciencias Biológicas y Agropecuarias, Universidad Autónoma de Yucatán, Mérida 97100, Mexico; 4 Instituto de Biotecnología y Ecología Aplicada, Universidad Veracruzana, Xalapa Enríquez, Veracruz 91090, Mexico; 5 Facultad de Ciencias Biológicas y Agropecuarias, Región Tuxpan, Universidad Veracruzana, Túxpan de Rodríguez Cano, Veracruz 92870, Mexico

**Keywords:** *Rickettsia*, companion animals, zoonoses, PCR, epidemiology, generalized linear model, risk ratio

## Abstract

**Simple Summary:**

Rickettsioses are relevant emergent and reemergent zoonoses in the Americas, including Mexico. Murine typhus caused by typhus group (TG) *Rickettsia* is prevalent in humans and their companion animals, such as dogs. This study found that 23.9% of the dogs (34/142) were infected by TG *Rickettsia* in Maxcanú, Yucatan (southeastern Mexico). Statistical analyses showed that reduced outdoor activities, age, sex and previous antiparasitic treatment are associated factors with less risk of TG *Rickettsia* infection in the sampled dogs. Monitoring and controlling these factors could help to restrict the enzootic transmission risk and prevent the potential zoonotic transmission.

**Abstract:**

The aim is to describe the Typhus group (TG) *Rickettsia* infection in dogs and to identify factors associated with this infection. We collected blood samples and gathered exposure and clinical data of 142 dogs from a rural community of Yucatan. The *Rickettsia* group was determined by semi-nested PCR. Generalized linear models with binomial error distribution were used to model the associated factors from the dog sample for risk ratio (RR) estimation. Thirty-four dogs (23.9%) showed molecular evidence of TG *Rickettsia* DNA. The multivariate model showed that mixed-breed dogs (RR = 0.06) and dogs that had received antiparasitic treatment (RR = 0.049) had a lower risk of getting infected, taking as reference the purebred group and the non-treated dogs, respectively. Looking at variable interactions, adult dogs without outdoor activities had a lower infection risk than puppies (RR = 0.26). Among dogs with antiparasitic treatment, females had a higher infection risk than male dogs (RR = 26.2). The results showed enzootic TG *Rickettsia* circulation in dogs of a rural community. The factors outdoor activities, age and previous antiparasitic treatment, as well as the clinical variables signs of hemorrhages and epistaxis, were associated with a less chance of natural infection in the studied dogs. Prevention and control of the enzootic transmission risk of TG *Rickettsia* should help to reduce the potential zoonotic transmission of this pathogen.

## 1. Introduction

The typhus group (TG) *Rickettsia* is integrated by *Rickettsia prowazekii* and *Rickettsia typhi,* which are relevant species for public health because they are the causative agents of epidemic typhus in humans and a neglected zoonosis known as murine typhus, endemic typhus or flea-borne typhus, respectively [1].

After an extensive analysis of TG *Rickettsia* species in Mexico, Sánchez-Montes et al. [1] showed that the most common *Rickettsia* species of this group occurring in dogs is *R*. *typhi*, whereas *R*. *prowazekii* occurs only in the human population. In this regard, murine typhus disease is widely distributed in countries of the Mediterranean region, Africa, Southeast Asia and North America, but is more important in coastal or urban areas with high densities of synanthropic rats (*Rattus rattus* and *R. norvegicus*) [2,3].

The clinical manifestations in infected people include fever, headache, myalgia and rashes, among others. This leads to the misidentification or habitual confusion of this disease with other febrile diseases such as the deadly Rocky Mountain spotted fever, Dengue fever, Zika fever, West Nile fever, leptospirosis, meningitis, syphilis and other less common diseases [3,4,5]. However, *R*. *typhi* infection in dogs is subclinical [6], and many studies provide no description of clinical signs [7,8,9,10]; except for some cases such as a recent description by Juhasz et al. [11] for a clinically ill dog with intermittent fever, lethargy, inappetence and lymphadenopathy, with molecular and serological tests showing infection by *R*. *typhi*. Nonetheless, dogs are asymptomatic carriers, according to several clinical and epidemiological studies [8,12,13].

The classical enzootic transmission cycle of murine typhus causal agent (*R*. *typhi*) occurs naturally between the synanthropic rats and the Oriental rat flea, *Xenopsylla cheopis*, in whose intestines it multiplies and is then excreted in faeces while it feeds, infecting the new susceptible host by contamination of the mucous membranes or auto-inoculation by skin abrasion [2,5,7]. However, in some regions of North America, an alternative cycle has been described, in which humans accidentally participate in the spreading of the enzootic cycle to dogs, cats, and peridomestic opossums through the common cat flea, *Ctenocephalides felis* [14,15,16,17] since this arthropod has a generalist feeding habit [5], which facilitates the transmission among various susceptible hosts [3].

Recently, studies in a rural locality in the State of Yucatan, Mexico, performed by Torres-Castro et al. [18,19,20], found that 90% of the people exposed to *Rickettsia* sp. infection (antibodies) were dog owners, and 70% had more than one dog in their household. There is also molecular evidence of two members of the spotted fever group (SFG) *Rickettsia* (*Rickettsia rickettsii* and *Rickettsia parkeri*) and a single member of the TG (*R*. *typhi*) circulation, raising questions about the risk of the dog population regarding *Rickettsia* infection.

Dogs are epidemiologically significant as sentinels of *R*. *typhi* circulation in both endemic and non-endemic areas, making them incidental hosts of the bacteria and temporary hosts of the transmitting fleas. Nevertheless, there are few global studies assessing the general epidemiological factors and clinical aspects of naturally infected dogs with murine typhus [8,9,10], mainly in rural settlements.

Since people from Maxcanú showed exposure to TG *Rickettsia* [18], and there is evidence of *R*. *typhi* infecting domiciliated dogs in the studied region [9,10,21], this research addresses the following questions: what is the occurrence of the TG *Rickettsia* natural infection in domiciliated dogs? Are there factors that help to predict the relative risk (RR) of TG *Rickettsia* natural infection in the canine population from the studied locality?

## 2. Materials and Methods

### 2.1. Study Site

This study was carried out in Maxcanú, a rural community located in the state of Yucatan, Mexico (20°24′–20°50′ N, 89°54′–90°21′ W), which has a maximum elevation of 100 m above sea level. The climate is tropical sub-humid with rain in summer; the average annual temperature ranges from 26 to 28 °C, with an average annual rainfall from 700 to 1100 mm. The predominant vegetation surrounding the community is tropical deciduous forest and its secondary stages. Land use is primarily for extensive and traditional agriculture, and some areas are used for livestock [22].

### 2.2. Collection of Clinical and Exposure Variables of Dogs

The research protocol and the informed consent letter were evaluated and approved by the Research Ethics Committee (CEI) of the *Centro de Investigaciones Regionales “Dr. Hideyo Noguchi”* (CIR) of the *Universidad Autónoma de Yucatán* (UADY) (Protocol number CIRB-07-2018), Merida, Mexico.

The study design was a cross-sectional epidemiological survey. From February to April 2019, several public outreach events were held to verbally inform the public about rickettsial diseases and to recruit participants for this study. The people who voluntarily accepted the invitation were visited by our work team and answered an epidemiological questionnaire with the exposure variables for each examined dog. Subsequently, a group of veterinary practitioners inspected each dog (general physical examination) for the data collection on clinical variables. The physical examination was performed with all necessary measures to protect the physical well-being of the dogs and the workgroup’s safety.

Data on physical exams, epidemiological questionnaire, and blood samples from dogs were collected during a single visit to each owner’s household. All studied dogs were manipulated after authorization and consent signatures by the owners, who were always present during the physical exam and blood collection.

The exposure variables collected in the epidemiological questionnaire were as follows: sex (male or female); age (puppy: under 1 year old, or adult: over 1 year old); body size (small: less than 5 kg, or large: more than 5 kg); hair length (short: less than 5 cm from the body to top, or long: more than 5 cm from the body to top); hair color (dark: more than 50% body, or light: more than 50% body); breed (purebred or mixed breed); ectoparasite infestation (no infestation, only ticks, only fleas or fleas and ticks); level of infestation (no infestation: zero ectoparasites, mild: 1 to 10 ectoparasites, or severe: more than 10 ectoparasites); anatomical site of the infestation (no infestation, one infested body site or several body sites); outdoor activities (if the dog accompanies or not the owners to carry out recreational activities, agricultural activities or hunting, regardless of the time or frequency); specific resting place (inside or outside the dwelling, regardless of the time or frequency); and social cohabitation with other dogs in the dwelling (more than one dog in the dwelling or not).

The clinical variables collected in the physical examination included vaccination (yes or no, without quantifying the number or type of vaccine, according to the information provided by the owners); antiparasitic treatments against ectoparasites and endoparasites, applied anytime in the 3 months before the day of the physical examination (yes or no, regardless of the doses or commercial brands, according to the information provided by the owners); fever (no (≤39 °C) or yes (>39 °C) by the use of a thermometer via the rectum); hemorrhages and epistaxis (no or yes if any sign in the skin or the mucosa, including oral, conjunctival, perianal and vulval in females); body condition (skinny; below 20% of the ideal body condition, ideal and overweight, regarding the breed [23]); skin lesions (no or yes if present in any body site, regardless of severity and distribution); bruising, petechiae and ecchymoses (no or yes, including oral mucosa, conjunctiva, perianal and vulvar mucosa in females); and other clinical signs such as nervous and locomotor disorders (yes or no) compatible with *Rickettsia* infection as previously described [24,25,26,27].

### 2.3. Blood Sampling in Studied Dogs and Genomic DNA Extraction

During the visits to the dwellings, after the physical examination, a sample of whole blood was collected directly from the saphenous vein (maximum 7 mL) with the help of a syringe and then placed in a sterile centrifuge tube (BD Vacutainer^TM^, Franklin Lakes, NJ, USA) with EDTA. Before bleeding, the area was cleaned with hydrogen peroxide and iodine to ensure that the blood was not exogenously contaminated with ectoparasite faeces during venipuncture.

The samples were preserved in hermetically sealed plastic portable refrigerators with refrigerants at approximately 4 °C during the fieldwork and subsequently transferred to the laboratory, where they were centrifuged at 1500 g at room temperature for 10 min. The white cell layer was collected and stored in a sterile 1.5-mL vial (Eppendorf^®^, Hamburg, Germany) at −80 °C.

Genomic DNA was extracted from the white cell layer, using the commercial Kit QIAamp DNA Mini Kit^®^ (QIAGEN, Hilden, Germany) protocol “DNA Purification from Liquids and Fluids”, following the manufacturer’s specifications. The DNA was quantified with the help of a spectrophotometer NanoDrop 2000^®^ (Thermo Scientific^®^, Waltham, MA, USA).

### 2.4. Molecular Identification of Rickettsia DNA

*Rickettsia* DNA molecular identification was performed by a multiple semi-nested polymerase chain reaction (snPCR), which allowed differentiation between the SFG and TG *Rickettsia* [28] by amplifying fragments of the *sca5* gene [29]. The primers, reagent concentrations and thermocycler conditions were the same as those described by Choi et al. [28]. The snPCR enables determining the infective *Rickettsia* group: bands of 420 base pairs (bp) for species belonging to the SFG and 237 bp for species belonging to the TG [10,28]. In all PCRs, the DNA of *Rickettsia conorii* was used as a positive control. The negative control was the reaction mixture (primers plus reagents) without DNA [10,18,19,20].

Electrophoresis was performed in 10% polyacrylamide gels (Sigma-Aldrich Products^®^, Taufkirchen, Germany) deposited in vertical cameras (Bio-Rad Laboratories^®^, Hercules, CA, USA) and stained with 1.1 molar silver nitrate. For the final visualization and recording of the results, a UV light transilluminator was used (Hoefer^®^, Holliston, MA, USA).

### 2.5. Statistical Analysis

All exposure variables collected in the epidemiological questionnaire, the clinical variables obtained in the physical examination and the results of the laboratory tests (snPCR) were captured in a digital database using the Excel^®^ software (Microsoft^®^, Bellevue, WA, USA). All categorical variables were analyzed with descriptive statistics. The *Rickettsia* TG infection frequency in the studied dog population was determined according to the results of the snPCR, and 95% confidence intervals were estimated with the Clopper-Pearson procedure [30,31].

A generalized linear model (GLM) with binomial error distribution and log link function, using the backward stepwise selection procedure, was used to select the predictor exposure variables for TG *Rickettsia* infection in dogs, accounting for those variables with a *p* value < 0.15 [32]. Subsequently, we explored the interactions between the most informative variables stratifying the analysis. Different models were constructed using Akaike’s criterion for model selection. The statistical significance for the modelled variables was set with a *p* < 0.05 value to estimate risk ratios (RR) and 95% confidence intervals. Statistical analyses were executed in R version 3.6.0 [33].

## 3. Results

A total of 142 dogs living in 58 households from Maxcanú were included in the sample. The frequencies of all categorical variables accounted from the survey are presented in Table 1. As can be seen, 76 (53.5%) dogs were males and 66 (46.5%) were females. According to age, 51 (35.9%) were puppies and 91 (64.1%) were adults; the average age for the studied dog population was 3.1 years, with a range from 2 months to 12 years. However, some owners were unable to refer the exact age of their dog. Regarding the body size, 45 (31.7%) dogs were small and 97 (68.3%) were large.

Most of the studied dogs had long (woolly) hair (92, 64.8%), and dark coloration was the most frequent (79, 55.6%). The dominant breed was the mixed breed (137, 96.5%). Eighteen individuals (12.7%) had no ectoparasites, and 56 (39.4%) had an infestation with fleas and ticks. The severe infestation level was the most frequent (82, 57.7%), followed by mild (42, 29.6%). In total, 106 (74.6%) dogs had an infestation in several body sites. Most of the studied dogs did not join their owners for outdoor activities (98, 69%) and did not have a specific place to sleep or rest inside the house (128, 90.1%). Finally, 119 (83.8%) dogs lived in the same house or in social convivence with at least one dog. None of the examined dogs were sterilized.

Regarding the clinical-oriented variables, in 84 (59.2%) studied dogs, the owners indicated that at least one vaccine was applied (mainly against the Rabies virus), and 41 (28.9%) had received antiparasitic treatment at least 3 months before blood sampling (ivermectin in different commercial presentations and doses, according to owners); 87 (61.3%) dogs had a skinny body condition. The most frequent clinical signs observed in the physical examination were skin lesions (42, 29.6%), followed by bruising, petechiae or ecchymosis (27, 19.0%), fever (25, 17.6%), other clinical signs related to *Rickettsia* infection (24, 17%), signs of hemorrhages and epistaxis (22, 15.5%) and pruritus (19, 13.4%).

The snPCR showed that 34 (23.9%) (95% CI = 17.2–31.8) of the studied dogs were positive for *Rickettsia* species belonging to the TG.

Of the 34 infected dogs, 18 (52.9%) were female and 16 (47.1%) were male. Regarding the ages, 15 (44.1%) were puppies and 19 (55.9%) were adults. The large-body size was most frequent in the infected dogs (23, 67.6%). Twenty-two dogs (64.7%) had long (woolly) hair, and 20 dogs (58.8%) had dark hair. The majority were mixed breeds (28, 90.3%), whereas the remaining three individuals were two Chihuahuas (5.8%) and a Golden Retriever (2.9%).

Tick and flea co-infestations were most frequent in the infected dogs (14, 41.2%), followed by only flea or only tick infestation (8, 23.5%). The severe infestation level was the most frequent (20, 58.8%). Similarly, most of the infected dogs had ectoparasites in several anatomical areas (27, 79.4%). Twenty-seven (79.4%) infected dogs did not join their owners in outdoor activities, 31 (91.2%) did not have a resting place inside the household, and 30 (88.2%) cohabitated with at least one other dog in the household.

Regarding clinical variables, 17 (50%) infected dogs had no vaccination, and 25 dogs (73.5%) had not received any antiparasitic treatments. Seven (20.6%) had a fever and three (8.8%) had signs of bleeding (epistaxis, hemorrhage, among others). In the infected dogs, the most frequent body condition was skinny (19, 55.9%), followed by ideal (15, 44.1%). Skin lesions, pruritus and petechiae or ecchymosis occurred in eight (23.5%), six (17.6%) and six (17.6%) dogs, respectively. Finally, three (8.8%) infected dogs showed other clinical but uncommon signs compatible with *Rickettsia* infection.

The GLM analysis showed that the most informative associated factors, sex, age, breed and outdoor activities, together with clinical variables, antiparasitic treatment at least 3 months before the sampling, vaccination, as well as hemorrhages and epistaxis, were statistically significant (*p* < 0.15) and were included to find a less complex multivariate model (Table 1).

After fitting different stratified models with interactions between variables, the final model included the following variable combinations: outdoor activities by age, breed, vaccination and antiparasitic treatment according to the dog’s sex (male or female). The multivariate model showed that mixed-breed dogs (RR = 0.06) and dogs that had received antiparasitic treatment 3 months before sampling (RR = 0.049) had a lower risk of *Rickettsia* TG infection, taking as reference the group of purebred dogs and the non-treated dogs, respectively. Looking at the interaction of the variables in the final model, we estimated that adult dogs without outdoor activities were at lower risk of *Rickettsia* TG infection than puppies (RR = 0.26), and among dogs with antiparasitic treatment, females had a higher risk of *Rickettsia* TG infection than male dogs (RR = 26.2) (Table 2).

## 4. Discussion

Dogs are some of the most frequent hosts of the TG *Rickettsia*. Sánchez-Montes et al. [1], in a recent bibliographic review on the *Rickettsia* occurrence in Mexico, showed their contribution to the transmission cycles of different *Rickettsia* species as incidental or temporary hosts of arthropod vectors, such as ticks and fleas [9,21,34,35].

Dogs suffer from intermittent infestations with *C*. *felis* fleas [36], making them susceptible hosts to TG *Rickettsia* infection such as *R*. *typhi* [8], acquiring epidemiological relevance for the occurrence of murine typhus in human populations, particularly for people who usually live with them [7]. However, reports with PCR tests of the occurrence of *R*. *typhi* natural infection in these companion animals are scarce [8,9,10].

The frequency of infection with TG *Rickettsia* found in the studied dogs from Maxcanú was higher than that reported by Martínez-Ortiz et al. [9] in domiciliated dogs from Bolmay, Yucatan (5.5% vs. 21.8%). Additionally, only one *Rickettsia* group (TG) was found circulating in the dog population from Maxcanú. The variation in the infection frequency might be due to changes in flea vector population densities and host susceptibility [7,37] or the neglected living of dogs in rural localities of Yucatan [38,39]. When fleas have generalist feeding habits (such as *C*. *felis*), the contact with mammals such as domiciliated dogs facilitates the enzootic spread of the bacteria [8].

The TG *Rickettsia* transmission cycle mainly depends on the ecology and behavior of its vector populations [3,5]. In this context, one of the most common fleas that infest domiciliated dogs from the State of Yucatan is *C*. *felis* [40], which, in some endemic regions, is the primary vector and reservoir of *R*. *typhi* [15,41] as well as the most abundant ectoparasite inside households and their surroundings [42], increasing the chances of *Rickettsia* TG transmission in the domiciliated dogs [21].

Most of the studied dogs from Maxcanú did not have veterinary services such as antiparasitic treatments. This circumstance increases the chances of finding infestation with ectoparasites (including fleas) in these animals [43]. However, the findings of this study showed the significance of antiparasitic treatments in these companion animals as treated dogs displayed a lower risk of having a *Rickettsia* TG infection.

When we adjusted the multivariate model for sex, we found that treated female dogs had an increased risk of infection with TG *Rickettsia* than treated male dogs. This finding can be partially explained by the less pronounced roaming habits of the female dogs compared to male dogs, as documented by Muinde et al. [44], because of several factors such as mating, pregnancy and the care of the young. Regarding that, extensive roaming behavior is a factor that modifies the risk for rickettsial infection [45,46,47]. In this context, female dogs could be spending more time in the peridomicile area, where exposure to flea vectors is frequent, as evidenced by the infestation levels of other hosts of *C. felis* and murine typhus, such as synanthropic opossums and small rodents in several localities of Yucatan [48,49,50,51]. However, the dynamics among flea-dog interactions in the peridomiciles of households should be addressed in future studies to obtain further information about the peridomicile cycles of the TG *Rickettsia*.

The multivariate model showed that mixed-breed dogs had a lower risk of occurrence of TG *Rickettsia* infection, taking as reference the group of purebred dogs; nonetheless, this result should be taken with caution because of the total number (*n* = 5) of purebred dogs in the sample was low. Our findings show the need for a deeper understanding of the physiopathology of TG *Rickettsia* infection in controlled experimental studies with companion animals to achieve a better understanding of the possible signs of infection by TG *Rickettsia*.

Outdoor activities such as hunting, going with the owners to agricultural areas or roaming into the surrounding patches of sylvatic vegetation, are suggested to have an effect on infection chances in the studied dogs. Likewise, age was an informative variable for the occurrence of TG *Rickettsia* infection in the studied dogs, but only when adjusted by outdoor activities. Concerning the multivariate results, adult dogs with outdoor activities have a lower risk of TG *Rickettsia* infection. In the state of Yucatan, dogs are exposed at an early age (puppies) to infestations with fleas and ticks [38], which might help to explain the increase in the infection risk in this group. Age probably plays a conditioning role in the susceptibility and immunological memory response in animals against various infections [52] and seems to influence their levels of infestation with ectoparasites [53,54].

The clinical signs caused by the natural infection with TG *Rickettsia* are not fully described [8,11] and, as well as other rickettsial infections such as *R*. *rickettsii*, *R. felis* and *R*. *conorii*, are usually subclinical, hardly recognizable and can cause non-specific and highly variable clinical signs such as fever, diarrhea, anemia, weight loss and skin rashes. However, these infections are generally self-limiting or disappear with the correct antimicrobial treatment [11,24,25,26,27,34]. It is also important to consider that previous infections with other non-pathogenic *Rickettsia* species could favor an immunological memory against future infections, including pathogenic *Rickettsia* species [1,12].

Some of those observed clinical signs agree with the findings in some infected dogs from Maxcanú; however, hemorrhages and epistaxis showed a lesser chance of being related with infection by TG *Rickettsia* because those are clinical signs compatible with those caused by *Ehrlichia canis* [8,24,27,55]. This microorganism is widely distributed in dogs from urban and rural areas of Yucatan [56,57,58,59]. Likewise, some studied dogs from Maxcanú showed flea and tick infestations, enabling natural infection with *E. canis* since natural coinfection with etiological agents transmitted by ticks and fleas has been documented in domiciliated dogs [27,53].

The present study showed interesting findings on TG *Rickettsia*-infected dogs. However, the study’s design had some limitations. The first was the lack of a priori sample size calculation. The sample included only dogs whose owners voluntarily agreed to participate in our study. Second, therefore, the sample did not have specific selection criteria for tick or flea-borne diseases, making some of the recorded clinical signs broad for other infectious diseases. Addressing these constraints, we estimated a posteriori sample size to dimension the effect of the sample power with 138 dogs from an infinite population with 0.21 of observed prevalence for TG *Rickettsia* infection (empirical) and 0.05 precision, leaving 0.85 confidence for the study’s result. In this regard, the classical epidemiological approach [60] recommends at least 0.9 confidence for acceptable observational field epidemiological studies, and our sample size was slightly smaller than suggested (142 dogs). On the second constraint, we could not specifically address detailed selection criteria for sampled dogs since the dog population from the study site was very heterogeneous, as documented previously by several regional studies [9,38,43]. Nonetheless, future studies must address the described constraints to increase power and refine model estimations.

The detection of TG *Rickettsia* DNA in dogs from Maxcanú suggests that they could be sentinels for the detection of risk areas or future potential outbreaks of murine typhus in humans [12,13] due to its proximity to human populations [45,61,62]. The epidemiological monitoring of pathogens transmitted by ectoparasites (ticks and fleas) in domiciliated dogs could be outstanding to prevent and control their transmission to human populations [45,46,63].

## 5. Conclusions

The *Rickettsia* TG species circulated actively in the studied dogs from a rural community of Yucatan State, representing a remarkable veterinary health problem and a potential zoonotic risk to their owners and the community, given the high frequency of animals naturally infected and infested with ectoparasites. Infection by TG *Rickettsia* in the studied dogs was subclinical. The factors of outdoor activities, age and previous antiparasitic treatment, as well as the clinical variables signs of hemorrhages and epistaxis, were associated with less chance of natural infection in the studied dogs.

## Figures and Tables

**Table 1:** Frequency of typhus group (TG) *Rickettsia* detected by semi-nested polymerase chain reaction (*Sca5* gene) relative to clinical and exposure variables of dogs from Maxcanú, Yucatan, Mexico, and their resulting *p*-values from the generalized linear model (GLM) with backward stepwise selection of variables fitted for the association analysis.

Variables	Number of Dogs (%)	TG-Infected Dogs (%)	GLM *p*-Value
Exposure variables			
Sex			
Male	76 (53.5)	16 (47.1)	
Female	66 (46.5)	18 (52.9)	0.1366
Age			
Puppy	51 (35.9)	15 (44.1)	
Adult	91 (64.1)	19 (55.9)	0.0439
Body size			
Small	45 (31.7)	11 (32.4)	
Large	97 (68.3)	23 (67.6)	
Hair length			
Short	50 (35.2)	12 (35.3)	
Long	92 (64.8)	22 (64.7)	
Hair color			
Dark	79 (55.6)	20 (58.8)	
Light	63 (44.4)	14 (41.2)	
Breed			
Purebred	5 (3.5)	3 (8.8)	
Mixed breed	137 (96.5)	31 (91.2)	0.0885
Ectoparasite infestation			
No infestation	18 (12.7)	4 (11.8)	
Only ticks	25 (17.6)	8 (23.5)	
Only fleas	43 (30.3)	8 (23.5)	
Ticks and fleas	56 (39.4)	14 (41.2)	
Level of infestation			
No infestation	18 (12.7)	4 (11.8)	
Mild	42 (29.6)	10 (29.4)	
Severe	82 (57.7)	20 (58.8)	
Anatomical site of infestation			
No infestation	18 (12.7)	4 (11.8)	
One site	18 (12.7)	3 (8.8)	
Several sites	106 (74.6)	27 (79.4)	
Outdoor activities			
No	98 (69)	27 (79.4)	
Yes	44 (31)	7 (20.6)	0.0954
Specific resting place			
Outside the household	128 (90.1)	31 (91.2)	
Inside the household	14 (9.9)	3 (8.8)	
Social coexistence with other dogs			
No	23 (16.2)	4 (11.8)	
Yes	119 (83.8)	30 (88.2)	
Clinical variables			
Vaccination			
No	84 (59.2)	17 (50)	
Yes	59 (41.5)	17 (50)	0.0330
Antiparasitic treatments (< 3 months)			
No	101 (71.1)	25 (73.5)	
Yes	41 (28.9)	9 (26.5)	0.0847
Fever			
No	117 (82.4)	27 (79.4)	
Yes	25 (17.6)	7 (20.6)	
Hemorrhages and epistaxis			
No	120 (84.5)	31 (91.2)	
Yes	22 (15.5)	3 (8.8)	0.0708
Body condition			
Skinny	87 (61.3)	19 (55.9)	
Ideal	55 (38.7)	15 (44.1)	
Skin lesions			
No	100 (70.4)	26 (76.5)	
Yes	42 (29.6)	8 (23.5)	
Pruritus			
No	123 (86.6)	28 (82.4)	
Yes	19 (13.4)	6 (17.6)	
Bruising, petechiae and ecchymoses			
No	115 (81)	28 (82.4)	
Yes	27 (19)	6 (17.6)	
Other clinical signs ^1^			
No	118 (83)	31 (91.2)	
Yes	24 (17)	3 (8.8)	

^1^ Infrequent reported clinical signs in dogs infected with *Rickettsia*.

**Table 2:** Results of the multivariate stratified analysis to identify the association between exposure and clinical variables with the occurrence of the typhus group (TG) *Rickettsia* infection in domiciliated dogs from Maxcanú, Yucatan, Mexico.

Variables	Coefficients	Multivariate *p* (<0.05)	RR (95% CI)
Outdoor activities ^1^			
Yes	−18.38	0.99	1.04 *×* 10^−^^8^ (4.9 *×* 10^−^^250^–364.5)
Breed ^2^			
Mixed breed	−2.9	0.03	0.06 (0.002–0.67)
Antiparasitic treatment ^1^			
Yes	−3.01	0.03	0.049 (0.001–0.43)
Hemorrhages and epistaxis ^1^ Yes	−1.45	0.11	0.23 (0.03–1.12)
Vaccination ^1^ Yes	2.39	0.06	10.9 (1.23–251.8)
Interactions ^3^			
Adult without outdoor activities Adult with vaccination	−1.35 −1.78	0.04 0.19	0.26 (0.007–0.89) 0.16 (0.0006–1.9)
Female with antiparasitic treatment	3.27	0.02	26.2 (2.6–911)

^1^ The negative category was used as a reference variable. ^2^ Purebred used as a reference variable. ^3^ Puppy without outdoor activities, puppy without vaccination and male with antiparasitic treatment were used as a reference variable. Coefficients = multivariate binomial regression coefficients; RR (95% CI) = risk ratio with 95% confidence interval.

## Data Availability

Informed consent was obtained from all subjects involved in the study.
